# Universal masking is an effective strategy to flatten the severe acute respiratory coronavirus virus 2 (SARS-CoV-2) healthcare worker epidemiologic curve

**DOI:** 10.1017/ice.2020.313

**Published:** 2020-06-25

**Authors:** Jessica L. Seidelman, Sarah S. Lewis, Sonali D. Advani, Ibukunoluwa C. Akinboyo, Carol Epling, Matthew Case, Kristen Said, William Yancey, Matthew Stiegel, Antony Schwartz, Jason Stout, Daniel J. Sexton, Becky A. Smith

**Affiliations:** 1 Division of Infectious Diseases and International Health, Department of Medicine, Duke University School of Medicine, Duke University, Durham, North Carolina; 2 Duke Center for Antimicrobial Stewardship and Infection Prevention, Duke University Medical Center, Durham, North Carolina; 3 Division of Occupational and Environmental Medicine, Department of Community and Family Medicine, Duke University Medical Center, Durham, North Carolina; 4 Occupational and Environmental Safety Office, Laboratory Safety, Duke University and Health System, Durham, North Carolina

Atypical presentations of severe acute respiratory coronavirus virus 2 (SARS-CoV-2) infection along with its ability to be transmitted from asymptomatic and presymptomatic individuals pose unique infection prevention challenges.^1-5^ Universal masking policies requiring all healthcare workers (HCWs) to wear face masks while on hospital premises are believed to reduce the risk of transmission in healthcare environments by providing source control and decreasing the spread of SARS-CoV-2 virus-laden oral and nasal droplets from infected individuals. We implemented universal masking (of all HCWs) as a strategy to preserve our workforce and to protect patients by reducing the risk of SARS-CoV-2 transmission from HCW to HCW, from patient to HCW, and from HCW to patient during asymptomatic or presymptomatic exposures. We aimed to measure the effect of universal masking on coronavirus disease 2019 (COVID-19) acquisition within the healthcare setting.

## Methods

Duke Health consists of a tertiary care academic hospital, 2 community hospitals, 21,014 HCW, and more than 180 primary care and specialty clinic practices in 10 counties in North Carolina, providing approximately 70,000 inpatient hospitalizations and 2.4 million outpatient visits annually. We prospectively recorded incident SARS-CoV-2 infections among HCW across our healthcare system to determine the impact of universal masking on nosocomial acquisition of SARS-CoV-2 within this population. We defined HCW to include all staff working in the inpatient or outpatient healthcare setting, regardless of the provision of direct patient care. Incident cases of HCW-associated SARS-CoV-2 cases were reported to the hospital system’s infection prevention team by Employee Health (EH). A team of case tracers interviewed all HCW patients to review potential community and occupational exposures. Based on the interview findings, each case was adjudicated by a panel of the authors (JS, SL, CE, MC, KS, WY, MS, BS) into the following categories: community-acquired, healthcare-acquired, or an unknown acquisition route. Community-acquired SARS-CoV-2 cases were defined as HCWs who had an unmasked exposure to a known positive person such as a family member, friend, or coworker outside of the hospital for greater than 10 minutes at less than 6 feet. Healthcare-acquired SARS-CoV-2 cases were defined as a HCW who had an unmasked exposure for greater than 10 minutes at less than 6 feet to another HCW who was symptomatic and tested positive for SARS-CoV-2 or a HCW who had an exposure to a patient with a positive SARS-CoV-2 test and was either not wearing the Centers for Disease Control and Prevention (CDC) (https://www.cdc.gov/coronavirus/2019-ncov/hcp/guidance-risk-assesment-hcp.html) fully-recommended Personal Protective Equipment (PPE) or reported a breach in PPE.

We used negative binomial regression to compare the incidence rates of healthcare-acquired SARS-CoV-2 cases among Duke Health HCWs before and after institution of universal masking using a likelihood ratio test. We also compared incidence rates of healthcare-acquired SARS-CoV-2 to community incidence rates from local counties (i.e. Durham, Granville, Orange, Person, and Wake) in North Carolina.

## Results

From March 15, 2020 to June 6, 2020 we assessed all HCWs who tested positive for SARS-CoV-2. Based on the panel adjudication, 38% cases were community-acquired, 22% were healthcare-associated, and 40% did not have a clear source of acquisition. Of note, 80% of HCWs did not work on COVID-19 units.

Of the healthcare-associated cases, 70% were related to unmasked exposure to another HCW for more than 10 minutes less than 6 feet apart and 30% were thought to be secondary to direct care of SARS-CoV-2 positive patients.

**Fig. 1.**
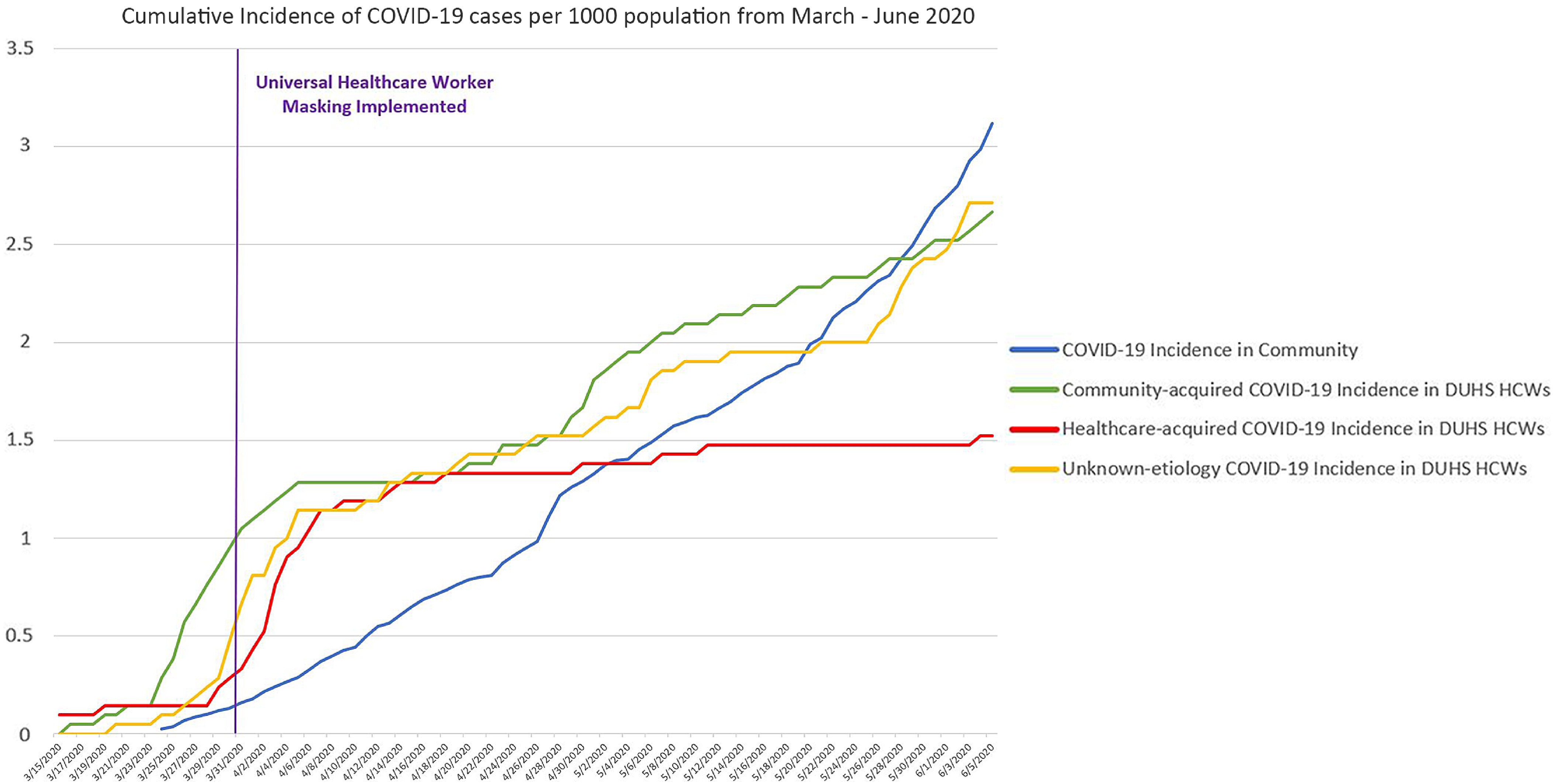
Cumulative incidence of positive SARS-2-CoV tests among healthcare workers stratified by community-acquired, healthcare-acquired, or unknown acquisition compared to local community cumulative incidence rates from Durham, Granville, Orange, Person, and Wake counties in North Carolina.

One week following the implementation of universal masking on March 31, 2020, we observed a significant decrease in the cumulative incidence rate of healthcare-acquired SARS-CoV-2 infections among HCWs (Figure 1) (LRT 4.38, p-value 0.03). The cumulative incidence rates in community-acquired cases and cases with no clear source of acquisition did not significantly change, however, and continued to mirror the cumulative incidence rates of SARS-CoV-2 in the communities surrounding Duke Health.

## Discussion

Universal masking of all HCWs significantly reduced the rate of healthcare-acquisition of SARS-CoV-2, thereby flattening the healthcare-associated SARS-CoV-2 epidemiologic curve in our healthcare system. HCWs with community-acquired SARS-CoV-2 or who had an unknown route of acquisition SARS-CoV-2 at the same incidence rate as other community members. We attribute the lower rate of healthcare-acquired infections in part to providing universal source control via masking, thereby mitigating the spread from asymptomatically infected or minimally symptomatic individuals. Mask etiquette, defined as wearing a mask at all times when physical distancing is not possible around anyone outside of your household contacts, limiting unmasked exposures indoors, and performing hand hygiene before and after touching the face mask, needs to be reinforced both inside and outside the workplace to help preserve the HCW workforce. Mask etiquette must also be performed alongside other infection prevention measures including following standard and transmission-based precautions, hand hygiene, physical distancing, and self-isolation coupled with immediate testing and contact notification when symptomatic. Finally, the recent changes to the CDC guidance that call for masking all inpatients and outpatients while direct care is provided and the addition of a face shield to our pandemic PPE outfit will hopefully lead to further reduction in healthcare-acquired SARS-CoV-2. HCWs will need ongoing reminders to follow recommended public health guidance to protect themselves from community acquisition of SARS-CoV-2 as the pandemic continues.
